# Subfamily Limoniinae Speiser, 1909 (Diptera, Limoniidae) from Baltic Amber (Eocene): The Genus *Elephantomyia* Osten Sacken, 1860

**DOI:** 10.1371/journal.pone.0117434

**Published:** 2015-02-23

**Authors:** Iwona Kania

**Affiliations:** Department of Environmental Biology, University of Rzeszów, Zelwerowicza 4, Rzeszów, Poland; University of Kansas, UNITED STATES

## Abstract

A revision of the genus *Elephantomyia* Osten Sacken (Diptera: Limoniidae) from Baltic amber (Eocene) is presented. Four species—*E*. *baltica* Alexander, *E*. *brevipalpa* Loew, *E*. *longirostris* Loew, and *E*. *pulchella* Loew—are redescribed and documented with photographs and drawings. In addition, two new species of the genus are described: *Elephantomyia bozenae* sp. nov., and *Elephantomyia irinae* sp. nov. All these fossil species are placed within the subgenus *Elephantomyia*. A key to the extinct species of *Elephantomyia* is provided, and the genus’ ecological pattern and evolutionary aspects are discussed.

## Introduction

The subfamily Limoniinae [1] of the most diverse dipteran family Limoniidae [1] comprises over 30 genera [2], of which 13 are well represented in the fossil record, including the genus *Elephantomyia* [3].

There are more than 130 extant species ascribed to the genus *Elephantomyia*, distributed across four subgenera: *Elephantomyia* (*Elephantomyia*) [3], *Elephantomyia* (*Elephantomyina*) [4], *Elephantomyia* (*Elephantomyodes*) [5], and *Elephantomyia* (*Xenoelephantomyia*) [6], [2]. These extant representatives of Diptera [7], occur mainly in Neotropical and Afrotropical regions [2]. In the Neotropics, the genus is represented by three subgenera, with 40 species belong to the typical subgenus, and one representative each of the subgenera *E*. (*Elephantomyina*) [4], and *E*. (*Xenoelephantomyia*) [6]. A similar number of species are reported from the Afrotropics, but all 37 species belong to a single subgenus: *E*. (*Elephantomyina*) [4]. In the Oriental, Australian, and Oceanian regions, *E*. (*Elephantomyodes*) [5] is the most species rich subgenus, although representatives of the typical subgenus are also found in all three regions.

Fossil representatives of the genus *Elephantomyia* are known from amber inclusions, mainly from Eocene Baltic amber, but none were assigned to a subgenus. Four species of *Elephantomyia* are known from the Baltic amber: *E*. *baltica* [8], *E*. *brevipalpa* [9], *E*. *longirostris* [9], and *E*. *pulchella* [9]. Three of these taxa—*E*. *brevipalpa* [9], *E*. *longirostris* [9], and *E*. *pulchella* [9]—were originally described as species of *Toxorhina* by Loew [10]. Osten Sacken [3] initially placed Loew’s fossils within the genus *Limnobiorhynchus*, but later [11] considered all of them part of his genus *Elephantomyia*. Although Scudder [12] claimed *Toxorhina* was a valid genus containing Loew’s fossil species and a single recent species, thereby rejecting their placement within *Elephantomyia*, a later revision [13] again ascribed these species to the genus *Elephantomyia* (Table 1). Handlirsch [14] listed Loew’s species within the genus *Toxorhina*, but listed the Osten Sacken [3] and Meunier [13] combinations as synonyms. Evenhuis [15] listed all Loew’s species in the genus *Elephantomyia*.

**Table 1:** List of species of the genus *Elephantomyia* from Baltic amber known so far and revised herein.

Subgenus	Species	The number of specimen	Material examined	Sex	Collection
*Elephantomyia*	*E*. *baltica*	282	Holotype	male	(GMUG)
	*E*. *bozenae* sp. nov.	MP/3338	Holotype	male	(ISEA PAS)
	*E*. *brevipalpa*	MB.J.337	Holotype	male	Coll. Berendt (NHMB)
		MP/3323	add. mat.	female	(ISEA PAS)
		161	add. mat.	male	Coll. Kutscher (GMUG)
	*E*. *irinae* sp. nov.	MP/3324	Holotype	male	(ISEA PAS)
		MP/3330	add. mat.	male	(ISEA PAS)
		MP/3331	add. mat.	male	(ISEA PAS)
		MP/3337	add. mat.	male	(ISEA PAS)
		250	add. mat.	male	Coll. Künow (GMUG)
	*E*. *longirostris*	MB.J.338	Holotype	male	Coll. Berendt (NHMB)
		1089–6	add. mat.	male	Coll. Ch. and H. W. Hoffeins
		MP/1627	add. mat.	female	(ISEA PAS)
		MP/3319	add. mat.	male	(ISEA PAS)
		MP/3322	add. mat.	male	(ISEA PAS)
		MP/3325	add. mat.	male	(ISEA PAS)
		MP/3328	add. mat.	male	(ISEA PAS)
		MP/3329	add. mat.	male	(ISEA PAS)
		MP/3333	add. mat.	male	(ISEA PAS)
		MP/3334	add. mat.	male	(ISEA PAS)
		19946	add. mat.	male	(ME PAS)
	*E*. *pulchella*	MB.J.336	Holotype	male	Coll. Berendt (NHMB)
		1195–5	add. mat.	male	Coll. Ch. and H. W. Hoffeins
		MP/3336	add. mat.	male	(ISEA PAS)

In addition to the species from Baltic amber, two species are also known from the Miocene Dominican amber: *Elephantomyia grata* [16], and an individual not identified to species level [17].

The discovery of a new *Elephantomyia* specimens from Baltic amber has allowed the description of two new extinct species within this genus. Further, this new research, which incorporates both previously known and new fossil materials, has enabled the revision of all *Elephantomyia* species from Baltic amber, and placement of these taxa into the subgenus *Elephantomyia*.

## Materials and Methods

### Specimens

The study herein is based on material from the collections: Institute of Systematic and Evolution of Animals, Polish Academy of Sciences (ISEA PAS) (15 specimens); Museum of the Earth, Polish Academy of Sciences, Warsaw (MEPAS) (1 specimen); University of Göttingen (GMUG) (1 specimen), Coll. Künow (GMUG) (1 specimen), Coll. Kutscher (GMUG) (1 specimen), Coll. Berendt, Natural History Museum Humboldt University, Berlin (NHMB) (3 specimens) and private collection of Christel and Hans Werner Hoffeins (2 specimens). Of the new species described herein, holotypes obtained from the Hoffeins’ collection will be deposited in Senckenberg Deutsches Entomologisches Institut (SDEI), Müncheberg, Germany.

### Nomenclature

The wing venation nomenclature is after Krzemiński [18], terminology for the male genitalia follows that of Ribeiro and Amorim [19], the terms “outer gonostylus” and “inner gonostylus” equal the terms “clasper of gonostylus” (branch II) and “lobe of gonostylus” (branch I) proposed by Ribeiro [20].

### Imaging

The specimens were studied using a Nikon SMZ 1500 stereomicroscope equipped with a Nikon DS-Fi1 camera. Drawings were produced using both specimens and photographs.

### Measurements

All measurements were taken with NIS-Elements D 3.0 software. The length of discal cell was measured from its basal edge to the point of connection of vein m-m with vein M_3_. The length of M_3_ is given from the wing margin to the point of connection of vein m-m with vein M_3_. The relations of rostrum, wing, and abdomen length are only given in those cases where the structures are not distorted. Chresonymy is used according to open nomenclature rules proposed by Matthews [21] and Bengtson [22] for the names of fossil taxa.

### Nomenclatural acts

The electronic edition of this article conforms to the requirements of the amended International Code of Zoological Nomenclature, and hence the new names contained herein are available under that Code from the electronic edition of this article. This published work and the nomenclatural acts it contains have been registered in ZooBank, the online registration system for the ICZN. The ZooBank LSIDs (Life Science Identifiers) can be resolved and the associated information viewed through any standard web browser by appending the LSID to the prefix “http://zoobank.org/”. The LSID for this publication is: urn:lsid:zoobank.org:pub: 546335C5–B194–4221–9F68-F20A3E8E2588. The electronic edition of this work was published in a journal with an ISSN, and has been archived and is available from the following digital repositories: PubMed Central, LOCKSS [author to insert any additional repositories].

### Systematic palaeontology

Order: Diptera Linnaeus, 1758

Family: Limoniidae Speiser, 1909

Subfamily: Limoniinae Speiser, 1909

### Genus: *Elephantomyia* Osten Sacken, 1860

**Type species**. *Limnobiorhynchus canadensis* [23]: 684, *sensu* [3]: 221; by original designation [= *Elephantomyia westwoodi* [11]: 109, misidentification).

### Subgenus: *Elephantomyia* Osten Sacken, 1860

**Type species**. *Limnobiorhynchus canadensis* [23]: 684, *sensu* [3]: 221; by original designation [= *Elephantomyia westwoodi* [11]: 109, misidentification).

### Key to species of the genus *Elephantomyia* Osten Sacken, 1860 from Baltic amber

1. Wings longer than rostrum; relatively short vein Rs, the length of Rs at least three times of the length of the basal section of R_5_ …….……….………………………….. 2.
2. Wings as long as rostrum; length Rs only slightly longer than twice the length of the basal section of R_5_… … ….………………………………………….……. ***E*. (*E*.) *baltica***
3. Palpus elongate, 4-segmented, much longer than the glossal lobes; antennae 15-segmented; Rs distinctly shorter than R_2+3+4_ ………………………………………… 3.
4. Palpus shorter than half the length of the rostrum’s glossal lobes; antennae 14-segmented; Rs as long as, or slightly longer than R_2+3+4_ ……………………………………………………………………..……… ***E*. (*E*.) *brevipalpa***
5. D-cell distinctly elongate, narrow, approximately twice as long as wide; vein M_3_ as long as d-cell ………………………………………………….. ***E*. (*E*.) *bozenae*** sp. nov.
6. D-cell wide, length approximately 1.5 times width; vein M_3_ 1.5 times longer than d-cell ……………………………………………………………………………………… 4.
7. Rostrum not very elongate, shorter than abdomen, distinctly shorter than wing, only slightly longer than half wing length ………………………….… ***E*. (*E*.) *irinae* sp. nov**.
8. Rostrum elongate, as long as or longer than abdomen, only slightly shorter than wing length ………………………………………….…….………………………………….. 5.
9. Wing approximately 1.3 times rostrum length; cross-vein m-cu at half of d-cell length ………………………………………………………………….…….. ***E*. (*E*.) *pulchella***
10. Rostrum very elongate, wing approximately 1.2 times the rostrum length; cross-vein m-cu just after the fork of Mb into M_1+2_ and M_3+4_………………….…. ***E*. (*E*.) *longirostris***

### *Elephantomyia* (*Elephantomyia*) *baltica* Alexander, 1931

v* 1931 *Elephantomyia baltica* Alexander, p. 88.

1994 *Elephantomyia baltica* Alexander, 1931: 58 [sic!]: Evenhuis, p. 69.

**Material examined**. Holotype: No. 282 (male), Coll. University of Göttingen (GMUG).

### Diagnostic characters

*E*. (*E*.) *baltica* clearly differs from the other Baltic amber species of the genus *Elephantomyia* in its relatively short Rs vein. The length of vein Rs is only slightly longer than twice the length of the basal section of R_5_, in other species of this genus, the length of Rs is at least three times that of the basal section of R_5_. *E*. (*E*.) *baltica* also differs distinctly in the ratio between the wing, rostrum, and abdomen lengths: in *E*. (*E*.) *baltica*, the wing is as long as the rostrum, whereas in other species, the wing is distinctly longer than the rostrum. In *E*. (*E*.) *baltica*, the rostrum is longer than abdomen, whereas in *E*. (*E*.) *brevipalpa*, *E*. (*E*.) *irinae* sp. nov., and *E*. (*E*.) *pulchella*, the rostrum is shorter than, or as long as, the abdomen. Additionally, vein Rs in *E*. (*E*.) *baltica* is shorter than R_2+3+4_, in contrast to *E*. (*E*.) *brevipalpa*, where Rs is as long as or longer than R_2+3+4_. Moreover, *E*. (*E*.) *baltica* has an elongate palpus, much like the other Baltic amber species of the genus *Elephantomyia*, whereas the palpus in *E*. (*E*.) *brevipalpa* is very short, being less than half the length of the rostrum’s glossal lobes. In *E*. (*E*.) *baltica*, cross-vein m-cu is situated just after the fork of Mb into M_1+2_ and M_3+4_, whereas in *E*. (*E*.) *pulchella*, m-cu is situated at exactly half the length of the d-cell.

### Redescription

Body: brown, 9.5 mm long (without rostrum).

**Fig 1:**
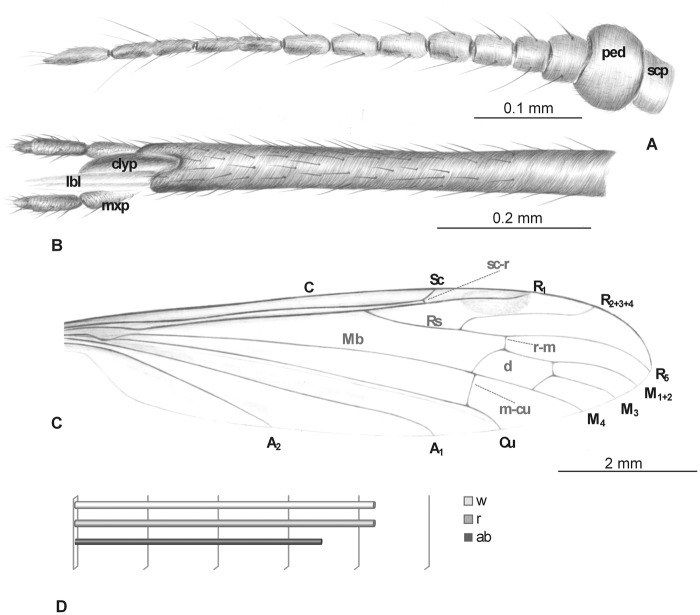
*Elephantomyia* (*E*.) *baltica* [8], No. 282 (male), holotype: A. antenna; B. apical part of rostrum with maxillary palps; C. wing venation; D. relation between the wing (w), rostrum (r), and abdomen (ab) lengths. Abbreviations: clyp—clypeus, lbl—labellum, mxp—maxillary palps, ped—pedicel, scp—scape.

Head: rostrum elongate, 8.5 mm long, as long as the wing, longer than abdomen (Fig. 1D). antenna (Fig. 1A) small; scape cylindrical; pedicel widened; basal flagellar segments short and crowded; last segments elongate, cylindrical, with two elongated setae on each flagellomere; palpus (Fig. 1B) elongate, 4-segmented, last segment short, other segments elongate and cylindrical; system of small microtrichia visible on all segments.

Wing (Fig. 1C): 8.5 mm long; pterostigma present, not darkened, oval, pale brown; vein Sc moderate length, ending opposite two-thirds Rs length; sc-r short, twice the distance from Sc tip; vein Rs gently arcuate, only slightly longer than twice length of basal section of R_5_, shorter than the length of R_2+3+4_; R_1_ ending approximately two-fifths length of R_2+3+4_; r-r (R_2_) atrophied; M_3_ one and half times longer than d-cell; cross-vein m-cu before d-cell mid-length, just after of fork of Mb; A_1_ and A_2_ almost straight.

Leg: tibial spurs not visible.

Hypopygium: not clearly visible.

Ovipositor: only male specimens known.

**Remarks**. The specimen is well preserved, but the number of segments of antenna is probably 14, but the flagellomeres are very crowded and the boundaries between individual flagellomeres is not clear.

### *Elephantomyia* (*Elephantomyia*) *bozenae* sp. nov. urn:lsid:zoobank.org:act:47F3BA11-F251–4BA2–8BE1–A85DEA1D0754

**Material examined**. Holotype: No. MP/3338 (male), Coll. Institute of Systematic and Evolution of Animals, Polish Academy of Sciences (ISEA PAS).

**Etymology**. The specific name is dedicated to the eminent biologist Bożena Szala, MSc.

### Diagnosis

*E*. (*E*.) *bozenae* sp. nov. is characterized by its elongate and narrow d-cell, which is approximately twice as long as wide; in *E*. (*E*.) *baltica*, *E*. (*E*.) *irinae* sp. nov., *E*. (*E*.) *longirostris*, and *E*. (*E*.) *pulchela*, the d-cell is shorter, being approximately 1.5 times longer than wide. In *E*. (*E*.) *bozenae* sp. nov., vein M_3_ is almost the same length as the d-cell, whereas M_3_ is longer than the d-cell in *E*. (*E*.) *baltica*, *E*. (*E*.) *irinae* sp. nov., *E*. (*E*.) *longirostris*, and *E*. (*E*.) *pulchella* sp. nov. Moreover, *E*. (*E*.) *bozenae* sp. nov. differs from other Baltic amber *Elephantomyia* species in the ratio of wing, rostrum, and abdomen lengths. In *E*. (*E*.) *bozenae* sp. nov., the rostrum is shorter than the wing, being slightly longer than half wing length, and longer than the abdomen. In contrast to *E*. (*E*.) *pulchella*, where m-cu is situated at exactly half of d-cell length, in *E*. (*E*.) *bozenae* sp. nov., m-cu is just before of half d-cell length. In *E*. (*E*.) *bozenae* sp. nov., Rs length is at least three times that of the basal section of R_5_, whereas in *E*. (*E*.) *baltica* the wing is as long as the rostrum, vein Rs is relatively short, and the length of vein Rs is only about twice the length of the basal section of R_5_. In *E*. (*E*.) *bozenae* sp. nov., Rs is shorter than R_2+3+4_, which contrasts to *E*. (*E*.) *brevipalpa*, where Rs is as long as R_2+3+4_ or longer. Moreover, the palpus in *E*. (*E*.) *bozenae* sp. nov. is elongate, unlike *E*. (*E*.) *brevipalpa*, where the palpus is very short, being shorter than half the length of the rostrum’s glossal lobes.

### Description

Body: brown, distal part of abdomen darker than rest of body, body 3.16 mm long (without rostrum).

**Fig 2:**
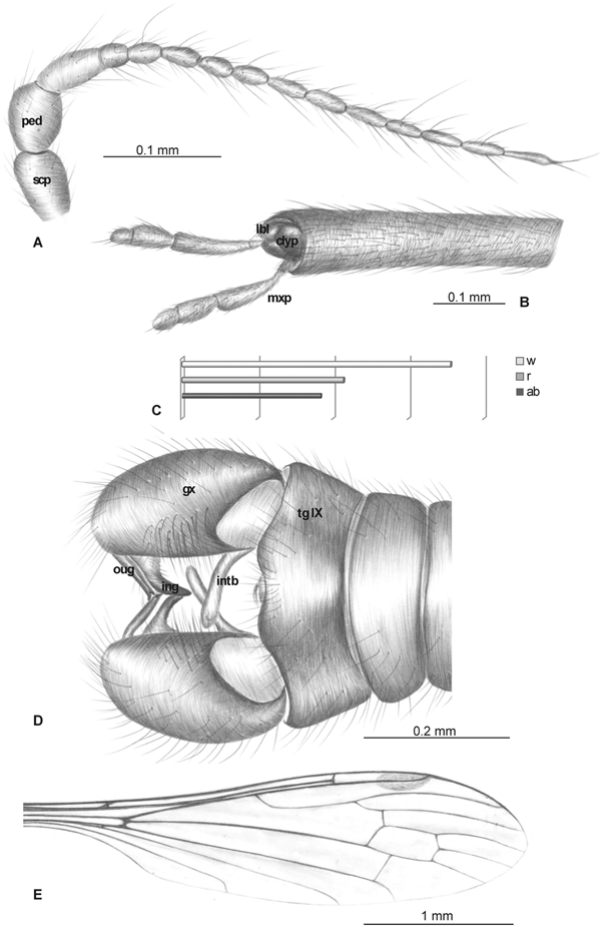
*Elephantomyia* (*E*.) *bozenae* sp. nov., No. MP/3338 (male), holotype: A. antenna; B. apical part of rostrum with maxillary palps; C. relation between the wing (w), rostrum (r), and abdomen (ab) lengths; D. hypopygium, dorsal view; E. wing venation. Abbreviations: clyp—clypeus, lbl—labellum, mxp—maxillary palps, ped—pedicel, scp—scape. Abbreviations: male terminalia: gx—gonocoxite; ing—inner gonostylus; intb—interbase; oug—outer gonostylus.

**Fig 3:**
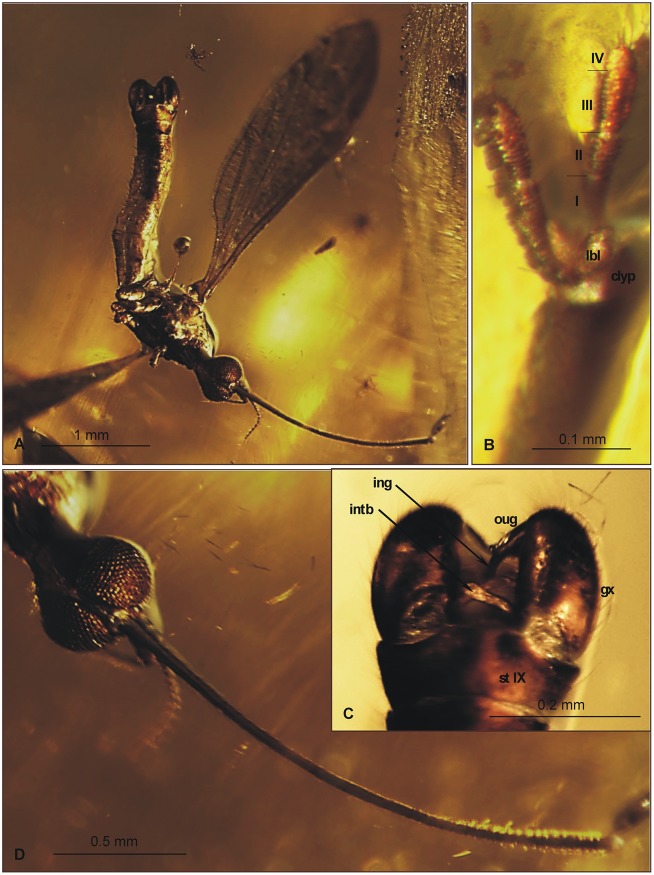
Morphology of *Elephantomyia* (*E*.) *bozenae* sp. nov., No. MP/3338, holotype: A. body, latero-ventral view; B. maxillary palpi; C. hypopygium, ventral view; D. head, ventral view. Abbreviations: clyp—clypeus, lbl—labellum, gx—gonocoxite, ing—inner gonostylus, oug—outer gonostylus, intb—interbase, st IX—sternite IX, I–IV—maxillary palps segments.

Head: rostrum elongate, 2.14 mm long, shorter than wing, ending just after half wing length, rostrum longer than abdomen (Fig. 2C). Antenna (Figs. 2A, 3D) small, 0.53 mm long, 15-segmented, flagellar segments crowded; scape cylindrical, widened; pedicel wide; first flagellomere elongate; second flagellomere very short, crowded with first flagellomere; flagellomeres 5–15 elongate; the last one flagellomere widened at apex; antennae with two elongated setae on each segment of antennae; palpus (Figs. 2B, 3B) elongate, 0.20 mm long, 4-segmented, the last segment short, other segments elongate. System of small microtrichia clearly visible on all segments.

Wing (Figs. 2E, 3A): 3.56 mm long, 0.86 mm wide; pterostigma present, not darkened, oval, pale brown; vein Sc elongate, ending opposite five-sixths Rs length; sc-r short, twice the distance from Sc tip; Rs slightly arcuate, at least three times basal section of R_5_, shorter than length of R_2+3+4_; R_1_ ending approximately half length of R_2+3+4_; r-r (R_2_) atrophied; M_3_ almost equal to d-cell length; cross-vein m-cu just before of d-cell mid-length; A_1_ almost straight, A_2_ slightly waved.

Leg: tibial spurs not visible.

Hypopygium (Figs. 2D, 3C): 0.37 mm, gonocoxite as in other *Elephantomyia* species, approximately twice as long as wide, with elongate, narrow, lobe-shaped interbase; outer gonostylus narrow, distinctly bifid at end, distal part curved externally; inner gonostylus slightly widened, strongly narrowed in apical part; directed into hypopygium.

Ovipositor: only male specimens known.

### *Elephantomyia* (*Elephantomyia*) *brevipalpa* (Loew, 1851)

1850 *Toxorhina brevipalpa* Loew, p. 37. (*nomen nudum*).

*. 1851 *Toxorhina brevipalpa* Loew, p. 400.

1860 (1859) *Limnobiorhynchus* [*brevipalpa*] Osten Sacken, p. 221.

1869 *Elephantomyia* [*brevipalpa*]: Osten Sacken, p. 106.

1894 *Elephantomyia brevipalpa* Osten Sacken [sic!] (*Toxorhina*): Scudder, p. 180.

1906 *Elephantomyia brevipalpa* (Loew, 1851): Meunier, p. 366.

1907 *Toxorhina brevipalpa* Handlirsch, p. 991.

1931 *Elephantomyia brevipalpa* (Loew, 1851): Alexander, p. 90.

1994 *Elephantomyia brevipalpa* Evenhuis, p. 69.

**Material examined**. Holotype: No. MB.J. 337 (male), Coll. Berendt (NHMB); No. MP/3323 (female), Institute of Systematic and Evolution of Animals, Polish Academy of Sciences (ISEA PAS); No. 161 (male), Coll. Kutscher, University of Göttingen (GMUG).

### Diagnostic characters

*E*. (*E*.) *brevipalpa* differs distinctly from other Baltic amber species of this genus in the characteristic morphology of its palpus. In *E*. (*E*.) *brevipalpa*, the palpus is very short, being less than half the length of the rostrum’s glossal lobes, whereas in other species, the palpus is relatively long. *E*. (*E*.) *brevipalpa* also differ from the other species redescribed and described here in the ratio between wing, rostrum, and abdomen lengths. In *E*. (*E*.) *brevipalpa*, the rostrum is shorter than the wing, being only slightly longer than half wing length, and shorter than abdomen. Moreover, in *E*. (*E*.) *brevipalpa*, vein Rs is as long as, or longer than, vein R_2+3+4_, in contrast to other species of this genus where Rs is distinctly shorter than R_2+3+4_. In *E*. (*E*.) *brevipalpa* Rs is more than three times the basal section of R_5_; in comparison, *E*. (*E*.) *baltica* has a relatively short Rs, only about twice the length of the basal section of R_5_. Cross-vein m-cu in *E*. (*E*.) *brevipalpa* is situated just after the fork of Mb into M_1+2_ and M_3+4_, whereas in *E*. (*E*.) *pulchella*, vein m-cu is half d-cell length. In *E*. (*E*.) *brevipalpa*, the d-cell is comparatively short and wide, being approximately one and half longer than wide, unlike *E*. (*E*.) *bozenae* sp. nov., where the d-cell is elongate and narrow, twice as long as wide. Moreover, vein M_3_ in *E*. (*E*.) *brevipalpa* is approximately one and half times longer than the d-cell, whereas in *E*. (*E*.) *bozenae* sp. nov., M_3_ is almost the same length as the d-cell.

### Redescription

Body: brown, 5.1 mm long (without rostrum) (female).

**Fig 4:**
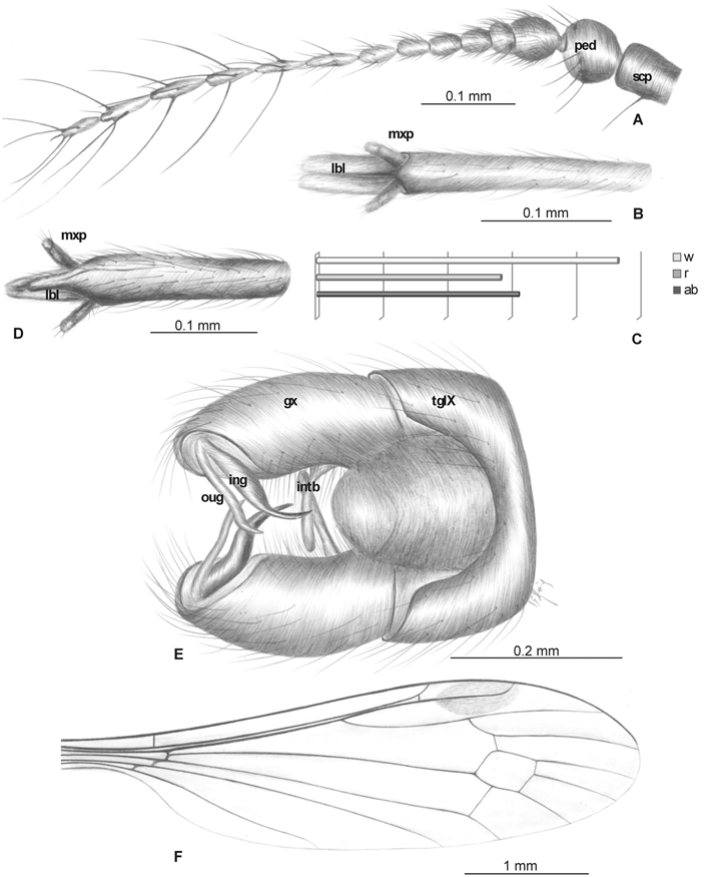
*Elephantomyia* (*E*.) *brevipalpa* [9]: A. No. 161 (male), antenna; B. No. MP/3323 (female), apical part of rostrum with maxillary palps (ventral view); C. relation between the wing (w), rostrum (r), and abdomen (ab) lengths; D. No. 161 (male), apical part of rostrum with maxillary palps (dorsal view); E. hypopygium; F. No. MP/3323 (female), wing venation. Abbreviations as in Fig. 2.

**Fig 5:**
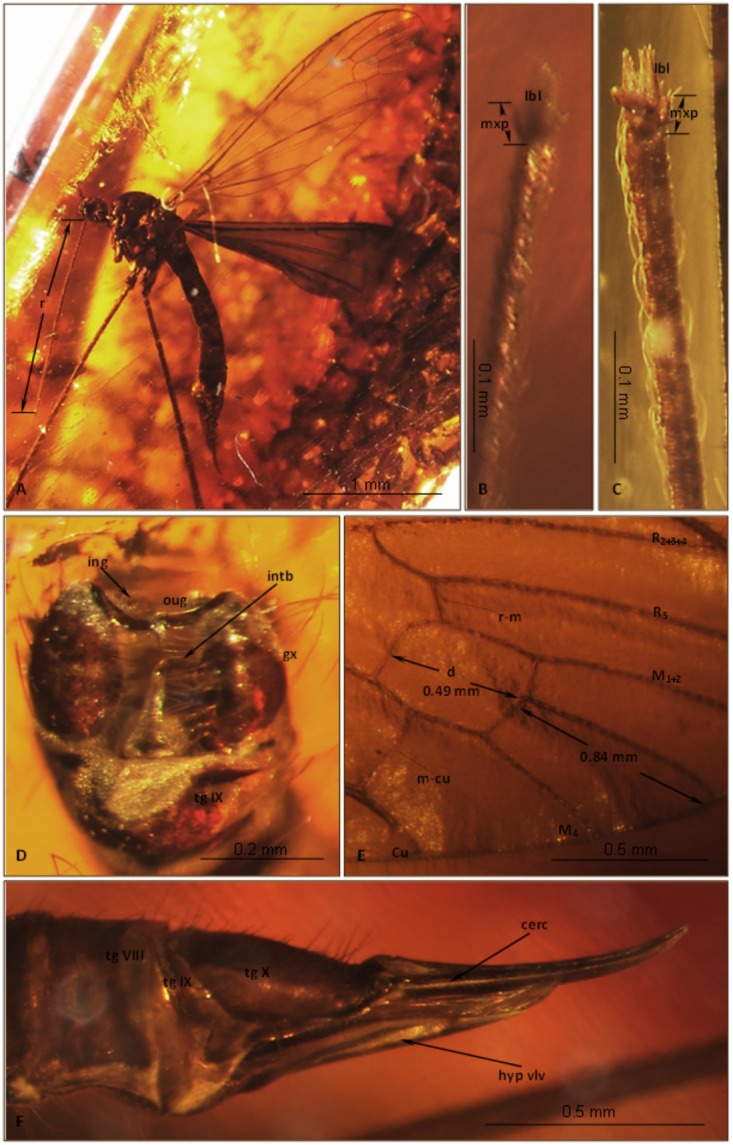
Morphology of *Elephantomyia* (*E*.) *brevipalpa* [9]: A. No. MP/3323, body, lateral view (female); B. No. MP/3323, maxillary palps (female); C–D. No. 161 (male), C. maxillary palps (male), D. hypopygium; E. No. MP/3323, wing apex (female); F. No. MP/3323, ovipositor. Abbreviations: gx—gonocoxite, ing—inner gonostylus, oug—outer gonostylus,, intb—interbase, r—rostrum, mxp—maxillary palps, lbl—labellum, cerc—cercus, hyp vlv—hypogynial valve, tg VIII—tergite VIII, tg IX—tergite IX, tg X—tergite X.

Head: rostrum elongate, 2.86 mm long (male), 2.86 mm long (female), shorter than wing, ending just longer than half wing length, longer than abdomen (male) (Figs. 4C, 5A). Antenna (Fig. 4A) relatively short, 14-segmented; scape elongated; pedicel widened; first flagellar segment widened; second flagellomere short; first flagellomeres crowded; flagellar segments 3–6 cylindrical and short; the last six segments cylindrical and elongate, with elongate setae that are much longer than the length of flagellomeres bearing them; flagellomeres 1–5 without elongate setae; flagellomeres 6–7 with three setae, not very elongate, shorter than segments bearing them; flagellomeres 8–11 with very elongate setae, much longer than segments bearing them; the last flagellomere with four very elongate setae; palpus (Figs. 4B, D, 5B, C) very short in both male and female, less than one half the length of rostrum’s glossal lobes; the last segment short, penultimate segment elongate and cylindrical.

Wing (Figs. 4F, 5A, E): 4.68 mm long, 1.18 wide (female); pterostigma present, not darkened, oval, pale brown; vein Sc moderate length, ending after half Rs length; sc-r short, twice distance from Sc tip; Rs slightly arcuate, at least three times length of R_5_ basal section, almost as long as, or longer than, R_2+3+4_; R_1_ short, ending approximately one-third length of R_2+3+4_; pterostigma base just after of Sc tip and before the bifurcation of Rs into R_1_, R_2+3+4_, and R_5_ (female); r-r (R_2_) atrophied; M_3_ approximately one and half times longer than d-cell; cross-vein m-cu just before d-cell mid-length; A_1_ almost straight, A_2_ slightly waved.

Leg: tibial spurs presented.

Hypopygium (Figs. 4E, 5D): 0.4 mm long, gonocoxite as in other species of the genus, approximately twice as long as wide, with elongate, narrow, lobe-shaped interbase; outer gonostylus narrow, not forked at the end, inner gonostylus widened in half of length, strongly narrowed in final one-third of its length; outer and inner gonostyles directed internally; aedeagus elongate.

Ovipositor (Fig. 5F): 1.09 mm long, tergite 9 narrow; tergite 10 large, hypogynial valves and cerci narrow, almost equal in length.

**Fig 6:**
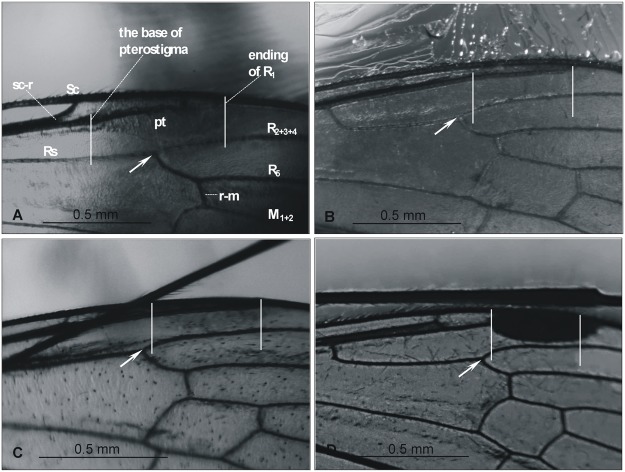
Differences in wing venation seen in *Elephantomyia* species from the Baltic amber: A. *E*. (*E*.) *brevipalpa*, No. MP/3323; B. *E*. (*E*.) *longirostris*, No. 1089–6; C. *E*. (*E*.) *irinae* sp. nov. No. MP/3324; D. *E*. (*E*.) *pulchella*, No. 1195–5.

**Remarks**. In contrast to other Baltic amber species of *Elephantomyia*, the pterostigma base in *E*. *brevipalpa* is distinctly shifted towards the base of wing, being just distal to the tip of Sc and proximal of the bifurcation of Rs into R_1_, R_2+3+4_, and R_5_ (Fig. 6A–C). However, this feature is only noted in the female wing, as details of the male wing are not observable due to poor preservation.

### *Elephantomyia* (*Elephantomyia*) *irinae* sp. nov. urn:lsid:zoobank.org:act:4540675A-AE8A-4766-B0D9–958BDACFF6E8

**Material examined**. Holotype: No. MP/3324 (male). Additional material: No. MP/3330 (male), MP/3331 (male), No. MP/3337 (male), Coll. Institute of Systematic and Evolution of Animals, Polish Academy of Sciences (ISEA PAS); No. 250 (male), Coll. Künow, University of Göttingen (GMUG).

**Etymology**. The specific name is dedicated to the eminent palaeoentomologist Dr. Irina D. Sukatsheva.

### Diagnosis

*E*. (*E*.) *irinae* sp. nov. differs from *E*. (*E*.) *longirostris*, *E*. (*E*.) *pulchella*, *E*. (*E*.) *bozenae* sp. nov., and *E*. (*E*.) *baltica* in the ratio of rostrum and wing lengths. In *E*. (*E*.) *irinae* sp. nov., the rostrum is distinctly shorter than wing, ending just after half wing length; in *E* (*E*.) *baltica*, the rostrum is as long as the wing, whereas in. *E*. (*E*.) *longirostris*, the wing is only one-fifth longer than the rostrum, and in *E*. (*E*.) *pulchella* the wing is one-third longer than the rostrum. In contrast to *E*. *bozenae* sp. nov., where the rostrum is longer than the abdomen, the rostrum in *E*. (*E*.) *irinae* sp. nov. is distinctly shorter than the abdomen. From *E*. (*E*.) *brevipalpa*, *E*. (*E*.) *irinae* sp. nov. differs in the length of palpus: where in *E*. *irinae* (*E*.) sp. nov. the palpus is elongate, in *E*. (*E*.) *brevipalpa* the palpus is very short, less than half the length of the rostrum’s glossal lobes. Moreover, in *E*. (*E*.) *irinae* sp. nov., the length of Rs is at least three times that of the basal section of R_5_, whereas in *E*. (*E*.) *baltica*, Rs is only about twice the length of the basal section of R_5_. Vein Rs in *E*. (*E*.) *irinae* sp. nov. is shorter than R_2+3+4_, in contrast to *E*. (*E*.) *brevipalpa*, where it is as long as, or longer than, R_2+3+4_. Cross-vein m-cu in *E*. (*E*.) *irinae* sp. nov. is situated after the fork of Mb into M_1+2_ and M_3+4_, but before half d-cell length; this is different from *E*. (*E*.) *pulchella*, where vein m-cu is situated at half d-cell length. In *E*. (*E*.) *irinae* sp. nov., the d-cell is rather short and wide, being one and half times as long as wide, whereas in *E*. (*E*.) *bozenae* sp. nov., the d-cell is elongate and narrow, and twice as long as wide. Moreover, vein M_3_ in *E*. (*E*.) *irinae* sp. nov. is approximately one and half times longer than the d-cell, but in *E*. (*E*.) *bozenae* sp. nov., M_3_ is almost the same length as the d-cell.

### Description

Body: dark, 9.5 mm long (without rostrum).

**Fig 7:**
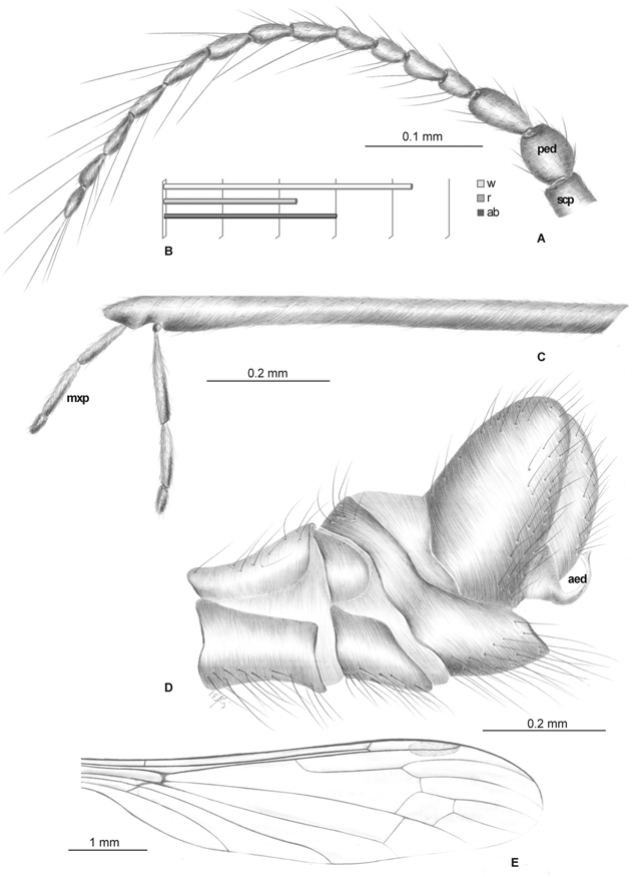
*Elephantomyia* (*E*.) *irinae* sp. nov., No. 250 (male), holotype: A. antenna; B. relation between the wing (w), rostrum (r), and abdomen (ab) lengths; C. apical part of rostrum with maxillary palps; D. hypopygium; E. wing venation. Abbreviations as in Fig. 2.

Head: rostrum not very elongate, 2.41–2.82 mm long, only slightly longer than half wing length, shorter than abdomen (Fig. 7B). Antenna (Fig. 7A) 15-segmented, small; scape elongated, cylindrical; pedicel widened; first flagellar segment elongate; second flagellar segment short; flagellomeres 5–15 elongate, crowded; flagellomeres 2–14 with three elongate setae; the last flagellomere with four elongate setae; setae on flagellomeres much longer than length of segments bearing them; palpus (Fig. 7C) elongate, 0.31–0.35 mm long, 4-segmented, the last segment short, other segments elongate and cylindrical.

**Fig 8:**
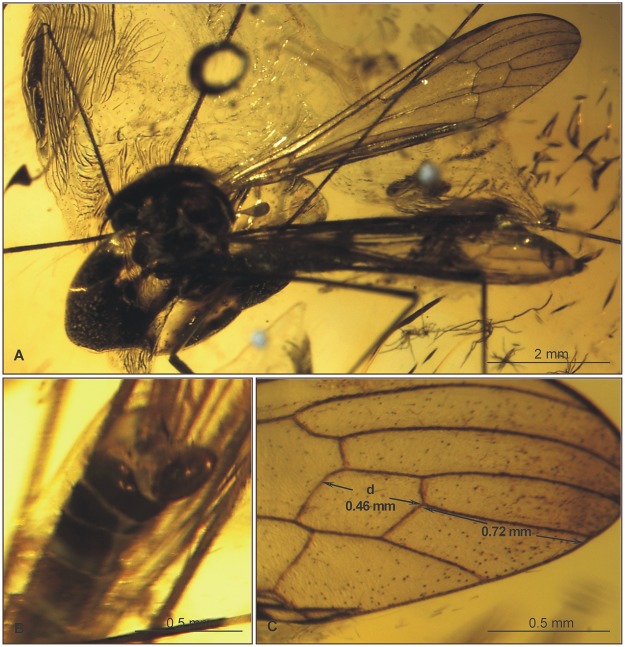
Morphology of *Elephantomyia* (*E*.) *irinae* sp. nov., No. MP/3324 (male): A. body; B. hypopygium; C. wing apex.

Wing (Figs. 7E, 8A, C): 3.49–8.5 mm long, 1.05–1.37 wide; pterostigma present, not darkened, oval, pale brown; vein Sc moderate length, ending opposite three-quarters Rs length; sc-r short, twice distance from Sc tip; vein Rs gently arcuate, Rs at least three times length of R_5_ basal section, shorter than half length of R_2+3+4_; R_1_ ending approximately half length of R_2+3+4_; r-r (R_2_) atrophied; M_3_ approximately one and half longer than d-cell; cross-vein m-cu before d-cell mid-length, about one-fourth its length beyond fork of Mb; A_1_ and A_2_ almost straight.

Leg: tibial spurs invisible.

Hypopygium (Figs. 7D, 8B): 0.55 mm, gonocoxite as in other species of the genus, approximately twice as long as wide, with elongate, narrow, lobe-shaped interbase; outer gonostylus narrow, distal part curved externally; inner gonostylus widened, directed into hypopygium; aedeagus elongate.

Ovipositor: only male specimens known.

### *Elephantomyia* (*Elephantomyia*) *longirostris* (Loew, 1851)

1850 *Toxorhina longirostris* Loew, p. 37 (*nomen nudum*).

* 1851 *Toxorhina longirostris* Loew, p. 400.

1860 (1859) *Limnobiorhynchus* [*longirostris*] Osten Sacken, p. 221.

1869 *Elephantomyia* [*longirostris*] Osten Sacken, p. 106.

1894 *Elephantomyia longirostris* Osten Sacken [sic!] (*Toxorhina*): Scudder: 180.

1906 *Elephantomyia longirostris* (Loew, 1851): Meunier, pp. 365–366.

1907 *Toxorhina longirostris* Handlirsch, p. 991.

p. 1931 *Elephantomyia longiostris* (Loew, 1851): Alexander, p. 90.

1994 *Elephantomyia longirostris* Evenhuis, p. 69.

**Material examined**. Holotype: No. MB.J. 338 (male), Coll. Berendt (NHMB); No. 1089–6 (male), Coll. Ch. and H. Hoffeins; No. MP/1627 (female); No. MP/3319 (male), No. MP/3322 (male), No. MP/3325 (male), No. MP/3328 (male), No. MP/3329 (male), No. MP/3333 (male), No. MP/3334 (male), Coll. Institute of Systematic and Evolution of Animals, Polish Academy of Sciences (ISEA PAS); No. 19946 (male), Coll. Museum of the Earth, Polish Academy of Sciences, Warsaw (MEPAS).

### Diagnostic characters

*E*. (*E*.) *longirostris* differs from other Baltic amber species of this genus in its very elongate rostrum, which is longer than the abdomen, and almost as long as the body length, being only one-fifth shorter than wing length. In *E*. (*E*.) *baltica*, the rostrum is longer than the abdomen and is equal in length to the wing; in *E*. (*E*.) *bozenae* sp. nov. and *E*. (*E*.) *brevipalpa*, the rostrum is longer than the abdomen but ends only slightly distal of half wing length; in *E*. (*E*.) *irinae* sp. nov., the rostrum is shorter than the abdomen; and in *E*. (*E*.) *pulchella* the rostrum is one-third shorter than the wing, whereas in *E*. (*E*.) *irinae* sp. nov., the rostrum ends just distal of half wing length. Although in *E*. (*E*.) *baltica* the length of vein Rs is only about twice the length of the basal section of R_5_, in *E*. (*E*.) *longirostris*, Rs is at least three times the length of the basal section of R_5_. In *E*. (*E*.) *longirostris* sp. nov. vein Rs is shorter than R_2+3+4_, which contrasts to *E*. (*E*.) *brevipalpa*, where Rs is as long as, or longer than, R_2+3+4_. Moreover, *E*. (*E*.) *longirostris* differs from *E*. (*E*.) *brevipalpa* in palpus morphology: in *E*. (*E*.) *longirostris*, the palpus is elongate, whereas in *E*. (*E*.) *brevipalpa* the palpus is very short, being shorter than half the length of the rostrum’s glossal lobes. Cross-vein m-cu in *E*. (*E*.) *longirostris* is positioned shortly after of the fork of Mb into M_1+2_ and M_3+4_, different from in *E*. (*E*.) *pulchella*, where m-cu is at exactly half d-cell length. In *E*. (*E*.) *longirostris*, the d-cell is comparatively short and wide, being approximately one and half times longer than wide, whereas in *E*. (*E*.) *bozenae* sp. nov., the d-cell is elongate and narrow, being twice as long as wide. Moreover, vein M_3_ in *E*. (*E*.) *longirostris* is one and half times longer than the d-cell, but in *E*. (*E*.) *bozenae* sp. nov. M_3_ is almost the same length as the d-cell.

### Redescription

Body: brown with elongate rostrum, 3.00–4.91 mm long (without rostrum).

**Fig 9:**
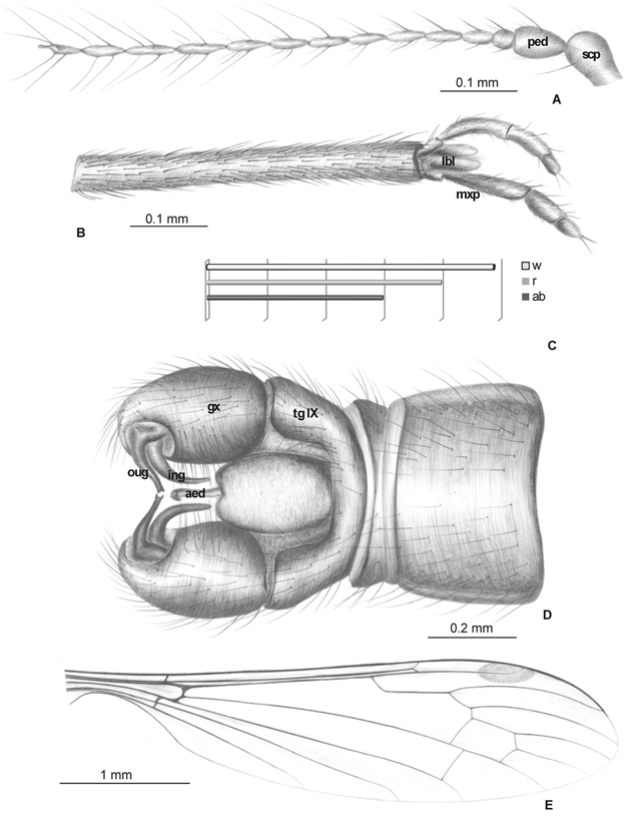
*Elephantomyia* (*E*.) *longirostris* [9], No. 1089–6 (male): A, antenna; B. apical part of rostrum with maxillary palps; C. relation between wing (w), rostrum (r), and abdomen (ab) lengths; D. hypopygium; E. wing venation. Abbreviations as in Fig. 2.

**Fig 10:**
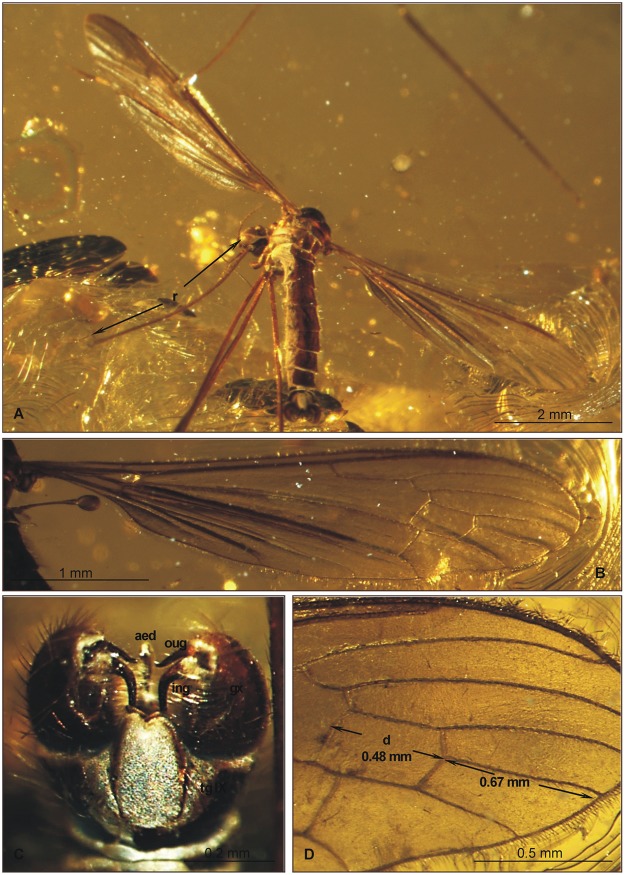
Morphology of *Elephantomyia* (*E*.) *longirostris* [9], No. 1089–6: A. body, latero-dorsal view; B. wing venation; C. hypopygium; D. wing apex. Abbreviations of male hypopygium features as in Fig. 9.

Head: rostrum 2.66–4.20 mm long, approximately equal to body length, longer than abdomen length, only one-fifth shorter than wing (Fig. 9C). Antenna (Figs. 9A, 10A) relatively short, 0.80–1.11 mm, 15-segmented; flagellar segments crowded; scape elongate; pedicel wide; flagellomeres 2–15 elongate; first flagellomere very short, crowded with previous segments; final segments elongate, narrowed at apex; antennae with four elongate setae on each flagellomere; setae much longer than length of segments bearing them; palpus (Fig. 9B) elongate, 4-segmented, final segment short, other segments elongate and cylindrical, system of small microtrichia clearly visible on all segments.

Wing (Figs. 9E, 10B, D): 4.23–8.5 mm long, 1.04–1.41 mm wide; pterostigma present, not darkened, oval, pale brown; vein Sc moderate length, ending after half Rs length; sc-r short, at end of Sc; vein Rs arcuate, at least three times length of basal section of R_5_, shorter than length of R_2+3+4_; R_1_ ending at approximately half length of R_2+3+4_; r-r (R_2_) atrophied; M_3_ approximately 1.5 times longer than d-cell length; cross-vein m-cu situated just after fork of Mb into M_1+2_ and M_3+4_; A_1_ almost straight, A_2_ slightly waved.

Leg: tibial spurs present.

Hypopygium (Figs. 9D, 10C): 0.50 mm, gonocoxite as in other species, approximately twice as long as wide, with elongate, narrow, lobe-shaped interbase; outer gonostylus narrow, distinctly bifid at end, distal part curved externally; inner gonostylus widened for basal half of its length, strongly narrowed in distal third, directed into hypopygium; aedeagus elongate.

Ovipositor: only male specimens known.

**Remarks**. The specimens No. K5100Z4080 (GMUG) and No. 87 (GMUG), reported as *E*. *longirostris* by Alexander [8], lack sufficient features to allow them to be clearly placed within this species. In these specimens, the rostrum is not very elongate, being about half of the wing length or shorter, and is shorter than, or as long as, the abdomen. Further study of these specimens is necessary to clarify their taxonomic status.

### *Elephantomyia* (*Elephantomyia*) *pulchella* (Loew, 1851)

1850 *Toxorhina pulchella* Loew, p. 37 (*nomen nudum*).

* 1851 *Toxorhina pulchella* Loew, p. 400.

1860 (1859) *Limnobiorhynchus* [*pulchella*] Osten Sacken, p. 221.

1869 *Elephantomyia* [*pulchella*] Osten Sacken, p. 106.

1894 *Elephantomyia pulchella* Osten Sacken [sic!] (*Toxorhina*): Scudder, p. 180.

1906 *Elephantomyia pulchella* (Loew, 1851): Meunier, p. 365.

1907 *Toxorhina pulchella* Handlirsch, p. 991.

1931 *Elephantomyia pulchella* (Loew, 1851): Alexander, p. 91.

1994 *Elephantomyia pulchella* Loew, 1851: Evenhuis, p. 69.

**Material examined**. Holotype: No. MB.J. 336 (male), Coll. Berendt (NHMB); No. 1195–5 (male), Coll. Ch. and H. W. Hoffeins; No. MP/3336 (male) Institute of Systematic and Evolution of Animals, Polish Academy of Sciences (ISEA PAS).

### Diagnostic characters

*E*. (*E*.) *pulchella* differs from all other species of the genus *Elephantomyia* known from the Baltic amber in the position of cross-vein m-cu, which in this species is located at exactly half d-cell length, whereas in other species of this genus, this cross-vein is situated just after the fork of Mb into M_1+2_ and M_3+4_ or just before the d-cell mid-length. *E*. (*E*.) *pulchella* also differs from the other fossil *Elephantomyia* species in the ratio of wing, rostrum, and abdomen length. In *E*. (*E*.) *pulchella*, the rostrum is one-third shorter than the wing, but is the same length, or slightly longer than, the abdomen. Moreover, in *E*. (*E*.) *pulchella* the length of Rs is at least three times that of the basal section of R_5_, whereas in *E*. (*E*.) *baltica*, vein Rs is relatively short, being only about twice the length of the basal section of R_5_. Additionally, Rs in *E*. (*E*.) *puchella* is shorter than R_2+3+4_, in contrast to *E*. (*E*.) *brevipalpa* where Rs is as long as, or longer than, R_2+3+4_. Moreover, the palpus is elongate in *E*. (*E*.) *pulchella*, much like other fossil species of the genus *Elephantomyia*, which differs from *E*. (*E*.) *brevipalpa*, where the palpus is very short, being less than half the length of the rostrum’s glossal lobes. In *E*. (*E*.) *pulchella*, the d-cell is comparatively short and wide, being approximately one and half times longer than wide; in *E*. (*E*.) *bozenae* sp. nov., the d-cell is elongate and narrow at twice as long as wide. Moreover, vein M_3_ in *E*. (*E*.) *pulchella* is approximately one and half times longer than the d-cell, whereas M_3_ is almost the same length as d-cell in *E*. (*E*.) *bozenae* sp. nov.

### Redescription

Body: brown, 3.42–3.63 mm long (without rostrum).

**Fig 11:**
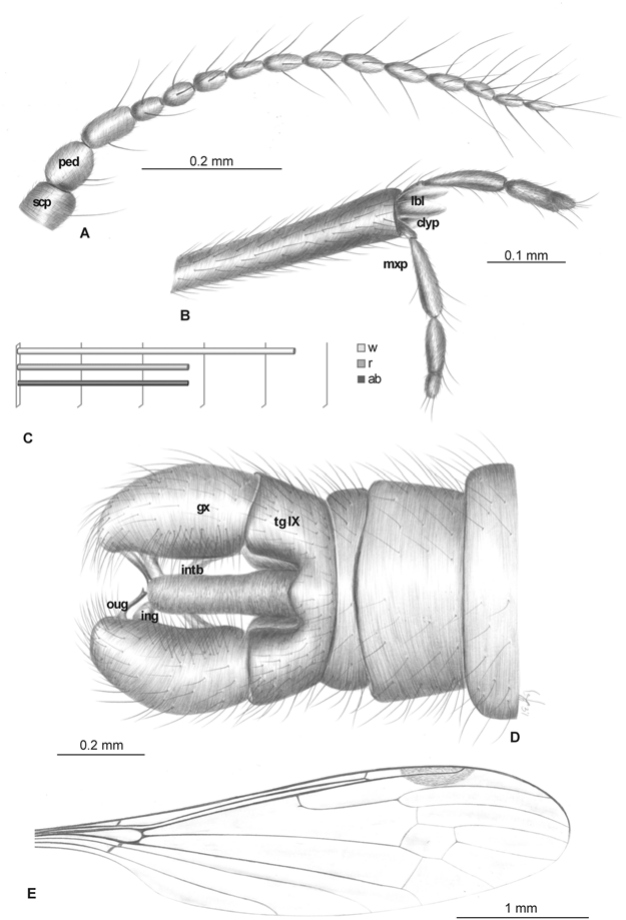
*Elephantomyia* (*E*.) *pulchella* [9], No. 1195–5 (male): A. antenna; B. apical part of rostrum with maxillary palps; C. relation between wing (w), rostrum (r), and abdomen (ab) lengths; D. hypopygium; E. wing venation. Abbreviations as in Fig. 2.

**Fig 12:**
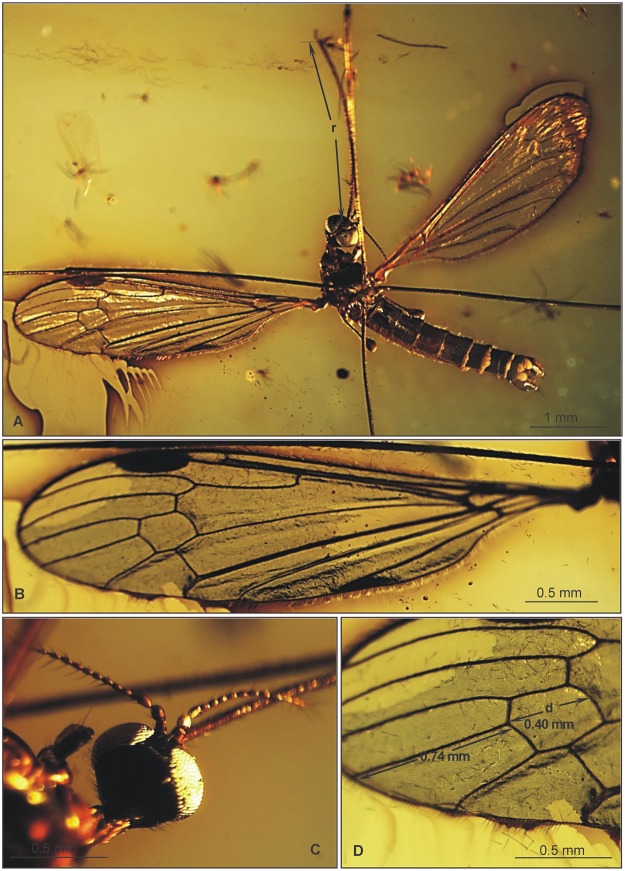
Morphology of *Elephantomyia* (*E*.) *pulchella* [9], No. 1195–5 (male): A. body, latero-ventral view; B. wing venation; C. head, dorsal view; D. apex of wing.

**Fig 13:**
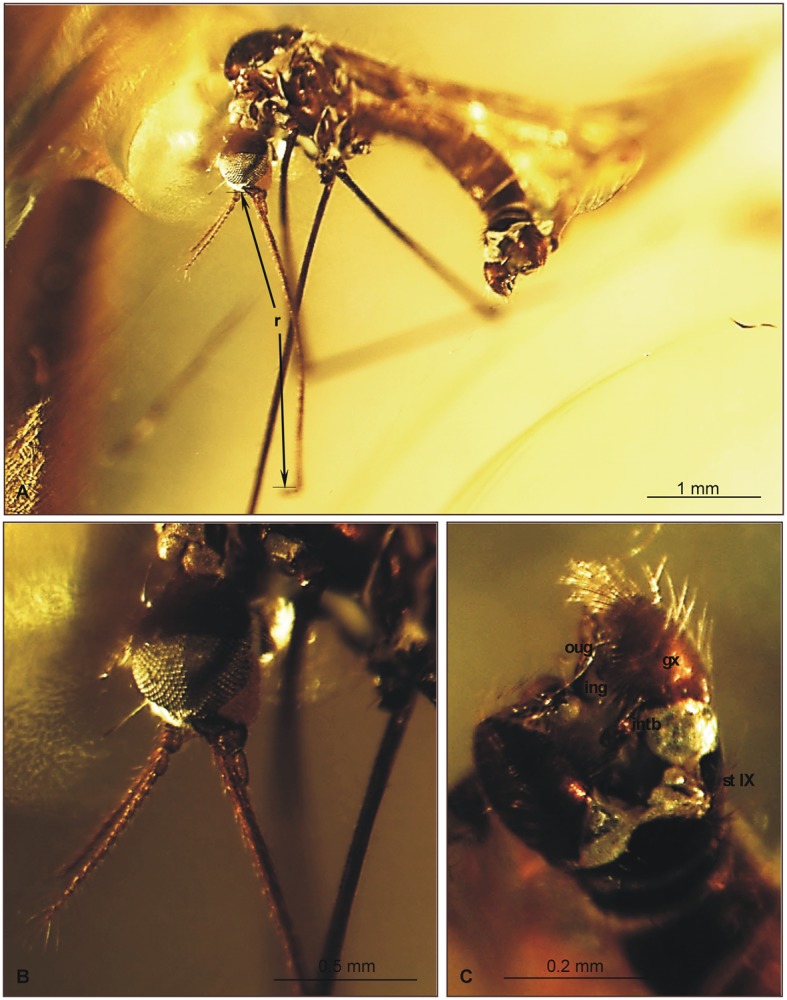
Morphology of *Elephantomyia* (*E*.) *pulchella* [9], No. MP/ 3336: A. body, latero-ventral view; B. head, lateral view; C. hypopygium, latero-ventral view. Abbreviations of male hypopygium features as in Fig. 11.

Head (Figs. 12C, 13B): head width 0.36–0.52 mm; rostrum elongate, 2.22–2.77 mm long, as long as or slightly longer than abdomen, one-third shorter than wing (Fig. 11C). Antenna (Figs. 11A, 12C, 13A, B) relatively short, 0.70–0.74 mm long, 15-segmented; flagellar segments crowded; scape elongate, cylindrical; pedicel wide; first flagellomere elongate, widened; second flagellomere short, widened, crowded with flagellomere 1; flagellomeres 3–6 short and widened; flagellomeres 7–15 elongate, cylindrical; final segments elongate, narrowed at apex; flagellomeres 1–5 with two elongate setae; flagellomeres 6–9 with three elongate setae; flagellomeres 10–13 with four elongate setae; elongate setae much longer than length of segments bearing them; palpus (Fig. 11B) elongate, 0.46 mm long, 4-segmented, final segment short, other segments elongate and cylindrical; system of small microtrichia clearly visible on all segments.

Wing (Figs. 11E, 12A, B, D): 3.8–4.50 mm long, 1.06 mm wide; pterostigma present, oval, pale brown; vein Sc moderate length, ending distal of half Rs length; sc-r short, at end of Sc; vein Rs distinctly arcuate, at least three times length of basal section of R_5_, shorter than length of R_2+3+4_; R_1_ ending at approximately half length of R_2+3+4_; r-r (R_2_) atrophied; M_3_ approximately one and half times longer than d-cell; cross-vein m-cu at exactly half d-cell length; A_1_ almost straight, A_2_ slightly waved.

Leg: tibial spurs present.

Hypopygium (Figs. 11D, 13C): 0.53 mm, gonocoxite as in other species, approximately twice as long as wide; interbase elongate and narrow, lobe-shaped; outer gonostylus narrow, bifid at end, distal part curved externally; inner gonostylus widened for basal half of its length, strongly narrowed for distal third of length; directed into hypopygium; aedeagus elongate.

Ovipositor: only male specimens known.

## Discussion

**Fig 14:**
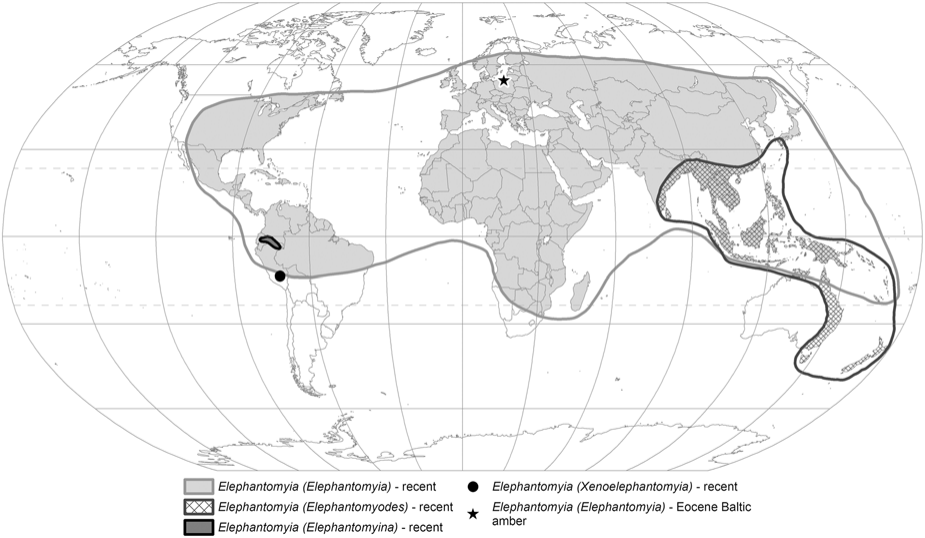
The distribution of recent and fossil *Elephantomyia*.

According to recent data, the first representatives of the genus *Elephantomyia* appeared in the Eocene, ~100 million years after the earliest representatives of the closely related genus *Helius* (*Helius lebanensis* [24], and *Helius ewa* [25], both from the Lower Cretaceous Lebanese amber). All of species of *Elephantomyia* known from Baltic amber can be placed within the nominative subgenus *Elephantomyia*, presently the most species-rich extant subgenus which is distributed worldwide. The other three extant subgenera—*Elephantomyina*, *Elephantomyodes*, and *Xenoelephantomyia*—are not presently represented in the fossil record, and are also rare in the extant fauna. Of these three subgenera, *Elephantomyina* is particularly rare, occur only in Ecuador and Peru, whereas *Xenoelephantomyia* is reported solely from Peru. These two subgenera each contain a single species: *Elephantomyia* (*Elephantomyina*) *supernumeraria* [26] and *Elephantomyia* (*Xenoelephantomyia*) *penai* [6]. The final subgenus, *Elephantomyodes*, is distributed in the Oriental, Australian, and Oceanian regions, and is represented by 32 extant species (Fig. 14). The fossil representatives of *Elephantomyia* known from the Baltic amber can be clearly placed within the subgenus *Elephantomyia*, and differ from species of the other subgenera particularly in regards to wing venation. In *Elephantomyina*, a strong supernumerary cross-vein connecting R_2+3+4_ and R_5_ occurs shortly before the tip of the latter, and r-m connects with Rs a short distance before its fork; in addition, the tibial spurs are absent in this subgenus [4]. *Elephantomyodes* differs from nominative subgenus in the lack of tibial spurs and the details of wing venation, particularly in having Rs in alignment with the basal section of R_4+5_ and R_2+3_, arising almost perpendicularly from the end of the sector [5]. In contrast to *Elephantomyia*, subgenus *Xenoelephantomyia* is characterised by a reduced anal field, with a single anal vein. Differences in the wing length to rostrum ratio are clearly observable among fossil representatives of the subgenus *Elephantomyia* known from the Baltic amber: In *E*. *baltica*, the rostrum is equal to the wing in length, whereas the rostrum is only slightly shorter than the wing in *E*. *longirostris* (1/7 shorter than the wing) or *E*. *pulchella* (1/3 shorter than the wing). *Elephantomyia* differs from the closely related genus *Helius* [27], [28] in the development of a poorly elongated rostrum, always shorter than half body length. In both *E*. *brevipalpa* and *E*. *irinae* sp. nov. we can observe that the rostrum is only slightly longer than half the wing length; these proportions can also be correlated to the length of abdomen, as in *E*. *brevipalpa* and *E*. *irinae* sp. nov. the rostrum is distinctly shorter than the abdomen, whereas in other representatives of this genus the rostrum is always longer than, or equal to, the abdomen length.

The elongate rostrum is also seen in the Cretaceous species *Helius ewa* [25], which can be considered closely related to *Elephantomyia*. Therefore, it could be assumed that this feature of an elongate rostrum appeared much earlier than the earliest *Elephantomyia* crane-flies [25], [28]. The development of such a structure has been related to feeding behaviour, as elongate, nectar-feeding mouthparts occur among many groups of dipterans, including the Limoniidae. In modern dipterans, the exploitation of the great variety of flower types and angiosperm taxa is permitted through a unique repertoire of sensory cues, in conjunction with modified mouthparts, and the presence of frequently large to holoptic compound eyes with stereoscopic, and probably colour, vision in advanced forms [29]. Therefore, it could be hypothesized that the appearance of the elongate rostrum in *Elephantomyia* and related forms reflected the diversification of flowers of various floral types, including the plesiomorphic ANITA-grade [30] which began to offer various ﬂoral rewards for pollination (brood sites, starchy food bodies, nectar, pollen, and heat as a resource). The diversification of *Elephantomyia* would have been promoted by further diversification and specialization of floral arrangements, their scent production, size, shape, colour, thermogenesis presence, insect rewards, and overall specialization [29]. These co-evolutionary processes probably took place during the late Cretaceous and early Palaeogene, when floral structures became more variable and elaborate, nectar was anatomically deployed in various positions within the flower, and the more ingenious pollination mechanisms developed [29], [31], [32]. However, knowledge of the biology and diversity of the extant species of *Elephantomyia*, and of all Limoniidae, is presently insufficient to provide detailed analyses of this idea.
